# The eyes and ears are visual indicators of attention in domestic horses

**DOI:** 10.1016/j.cub.2014.06.023

**Published:** 2014-08-04

**Authors:** Jennifer Wathan, Karen McComb

**Affiliations:** Mammal Vocal Communication and Cognition Research, School of Psychology, University of Sussex, Brighton, BN1 9QH, UK

## Abstract

Sensitivity to the attentional states of others has adaptive advantages [1], and in social animals, attending to others is important for predator detection, as well as a pre-requisite for normal social functioning and more complex socio-cognitive abilities [2]. Despite widespread interest in how social species perceive attention in others, studies of non-human animals have been inconclusive about the detailed cues involved [3]. Previous work has focused on head and eye direction, overlooking the fact that many mammals have obvious and mobile ears that could act as a visual cue to attention. Here we report that horses use the head orientation of a conspecific to locate food, but that this ability is disrupted when parts of the face (the eyes and ears) are covered up with naturalistic masks. The ability to correctly judge attention also interacted with the identity of the model horse, suggesting that individual differences in facial features may influence the salience of cues. Our results indicate that a combination of head orientation with facial expression, specifically involving both the eyes and ears, is necessary for communicating social attention. These findings emphasise that in order to understand how attention is communicated in non-human animals, it is essential to consider a broad range of cues.

## Teaser

Wathan and McComb show that horses use the head orientation of a conspecific to locate food, but that this ability is disrupted when the eyes and ears are covered up with naturalistic masks. Their results indicate that a combination of head orientation with facial expression s necessary for communicating social attention in horses.

## Main Text

Studies using naturalistic gaze-following paradigms have indicated that a wide range of animals, from crows to chimpanzees, follow the attention of conspecifics [2]. However, gaze is often used as a general term encompassing head orientation, eye direction and any other potential indicators. Consequently, it is difficult to establish exactly what cues are informative, and previous experimental work exploring this has focused on cues that humans use, in particular, head orientation and eye gaze [3,4], potentially overlooking a wealth of other available information. Animals with a different facial morphology — particularly those with large, mobile ears — may have other means of signaling.

**Figure 1:**
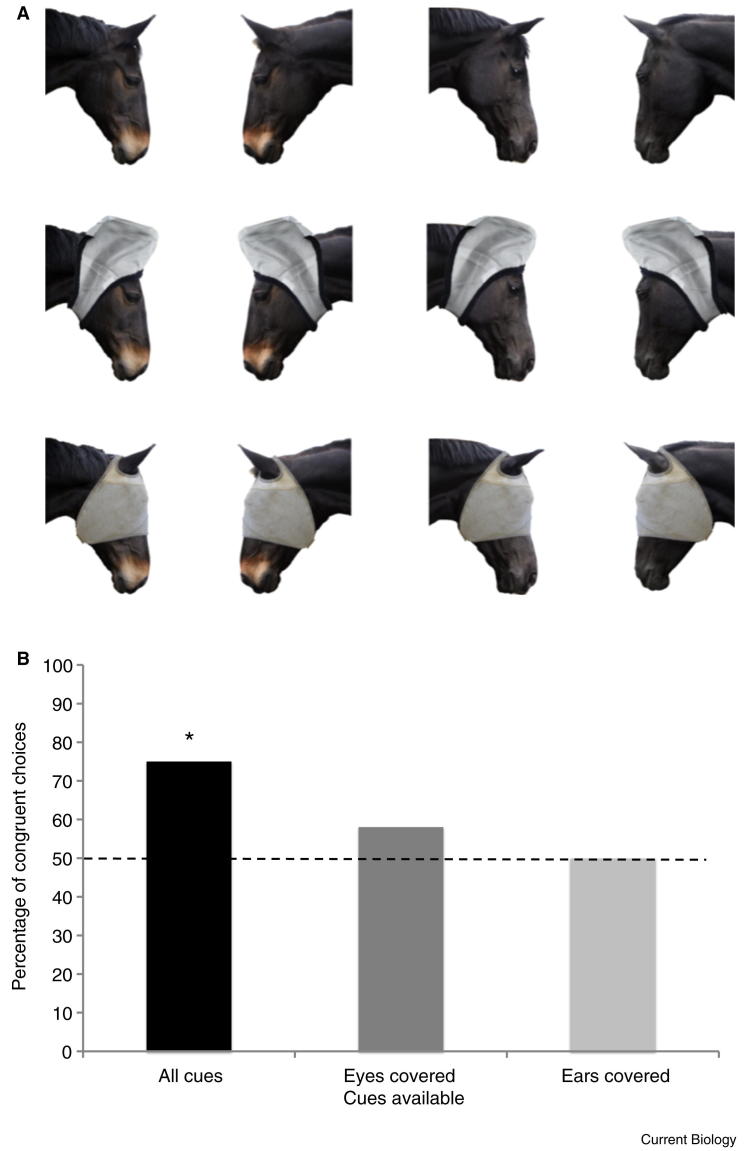
Illustration of stimuli and main results. Photographic stimuli including manipulations that incorporate fly masks to cover key internal features of the face. The photographs were reproduced at life size and used as the model in an object choice task to establish whether horses could use the head orientation and facial expression of a conspecific to locate hidden food. Both model horses are shown here in the three experimental conditions: all cues visible; ears covered; eyes covered. WG is the horse on the left of the image; MC is the horse on the right. (B) Percentage of horses choosing the congruent bucket for each condition. Asterisk: P < 0.05 (binomial probabilities, two tailed).

Horses are a prey animal with advanced social relationships [5–7], and within the domestic environment they often have parts of their faces covered by riding equipment or masks used for protection from flies. We used these masks, presented within photographic stimuli, to investigate whether horses were responsive to the attentional cues of another horse, and if so what areas of the face were important in providing information (Figure 1A, Supplemental Information and Figure S1).

In our experiment, horses were clearly sensitive to the attentional state of a conspecific and this influenced their decision about where to feed. When subjects viewed the unoccluded image of another horse looking at one of two buckets containing food, they were more likely to feed from the bucket congruent with the model (*n* = 24, *K* = 18, *P* = 0.02; Figure 1B). However, when either the eyes or ears were covered the choices of the participants dropped to chance levels (eyes: *n* = 24, *K* = 14, *P* = 0.54; ears: *n* = 24, *K* = 12, *P* = 1), suggesting these were both key areas informing the participants’ decisions.

The cues available — whole head visible, eyes covered, or ears covered — also influenced the time spent looking at the photographs (*F*(2,62) = 3.62, *P* = 0.03; see also the Supplemental Information). Planned comparisons revealed that horses looked for significantly longer when all the information was visible, compared to when the ears or the eyes were covered (*P* < 0.01). However, there was no difference in looking time when the ears were covered compared to when the eyes were covered, nor was looking time influenced by the identity of the model horse.

Additionally, more subtle effects were also apparent. Cues available, age, sex, testing centre, model horse viewed, and stimuli direction were entered as predictors in a logistic regression with feeding choice as the response variable (0 = choice incongruent with model; 1 = choice congruent with model; Supplemental Information and Table S1). This revealed a significant interaction, whereby sensitivity to the cues available differed according to the model horse viewed. Covering the eyes (Figure 1A) had less influence on subjects that viewed model MC than subjects who viewed model WG (see also Supplemental Information), potentially indicating that differences in the facial features or expression of the two models affected the salience of cues, and highlighting an interesting area for future research.

We also conducted additional presentations of single images as controls to verify that covering key parts of the face did not impede subjects’ recognition of the stimuli as depicting a horse. When subjects were allowed to view our horse stimuli at close range they showed similar reactions to all three conditions (all cues visible, eyes covered, ears covered), which were significantly different from their responses to appropriately matched control stimuli (phase-scrambled counterparts of the originals; Supplemental Information). Furthermore, subjects were more likely to approach the original stimuli and more likely to avoid the controls, as would be predicted if they were responding to photographs of horses versus novel objects (see details in Supplemental Information). We also took precautions to avoid the possibility of a ‘Clever Hans Effect’ occurring through incidental cueing by the experimenter. In particular, the experimenter was unfamiliar to the horse, kept ignorant of which stimuli would be presented and, cruicially, after the release point (when the choice was made) they stood facing away from the horse, so could not see the horse’s choice or provide any feedback (full details in Supplemental Information).

Our results provide the first evidence from an animal with laterally placed eyes that cues from this area convey important information. Eye gaze is difficult to isolate in animals with eyes positioned at an oblique angle, and it had been suggested that non-primates cannot use eye gaze independently of head orientation [2,4]. However, we demonstrate that the eyes do carry information, even when laterally placed in an animal far removed from the primate lineage. Horses, along with other ungulates, have a white sclera that is visible in various situations [6]. This plus other cues, such as dilation of the pupil and movement of the facial muscles surrounding the eye, could be informative of attentional state, as they are in humans [8].

Most significantly, our results demonstrate that animals with large, mobile ears can use these as a visual cue to attention. While anecdotal accounts of this exist in the literature (for example [6]) the potential role of the ears in signaling has been overlooked in previous experiments. In animals that have evolved a differently shaped face it is important to consider cues that humans do not have, and novel paradigms that incorporate these will be crucial in developing a full understanding of attentional mechanisms across species.

## Supplemental Information

Document S1. Experimental Procedures, Supplemental Results, One figure, and One table
