# Supplementary material for: The eyes and ears are visual indicators of attention in domestic horses
Source: Curr Biol. 2014 Aug 4;24(15):R677–9. doi: 10.1016/j.cub.2014.06.023 (PMC4123162; doi:10.1016/j.cub.2014.06.023)
Supplement: Document S1. Experimental Procedures, Supplemental Results, One figure, and One table [file mmc1.pdf]

Supplemental Information: The ears and eyes act as visual cues to attention in domestic horses

Jennifer Wathan and Karen McComb

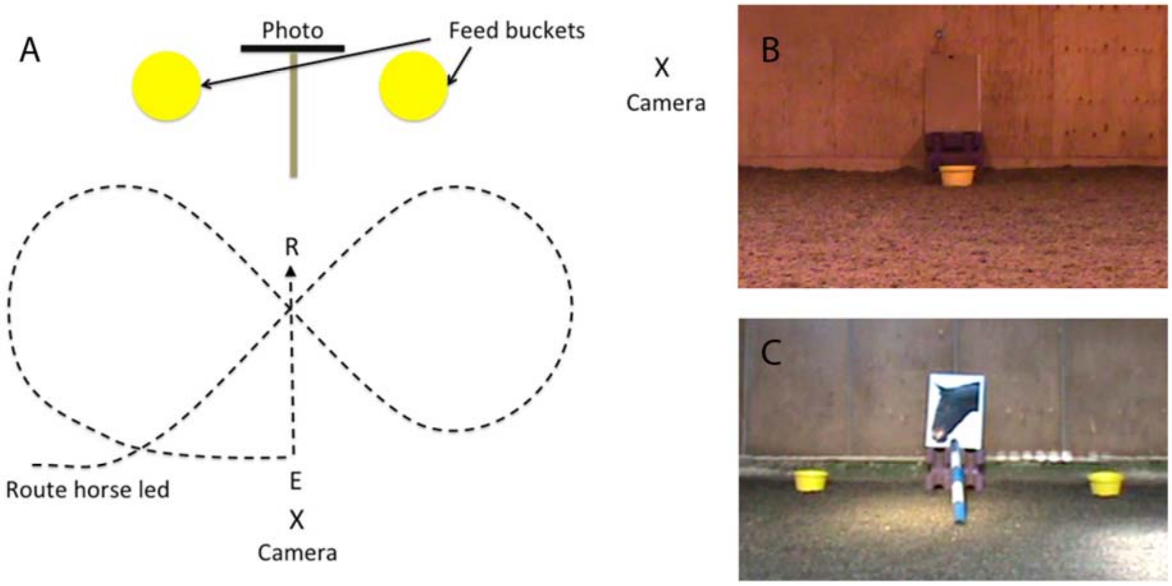

Figure S1. The experimental set up [related to experimental procedures and Figure 1A].

A. Diagram showing the testing area and route taken to the release point (R). All trials were conducted in an indoor area on the yard where the participant resided. Horses participated in one training trial (B) in which a bucket containing 20g of hard feed was placed against the wall. Horses were brought into the area and led in a figure of eight before being released (at point R) to approach the bucket and eat the food. If horses were happy to approach the bucket they then participated in one test trial (C) where a photographic stimulus was attached to the wall above a dividing pole raised at one end on a plastic block, with a bucket containing 20g hard food on either side (Stimulus shown in picture is WG, whole head visible). Horses were again walked in a figure of eight before being released at point R.

Table S1. Results of a linear regression analysis to determine the parameters affecting accuracy (0=incongruent choice, 1=congruent choice). The deviance value of a model is a measure of the goodness of fit. Deviance values in the table reflect the change in fit when significant variables are dropped and non-significant variables are added to the final model. These values are distributed approximately as Chi-squared (χ^2), with degrees of freedom (df) equivalent to the difference in the number of parameters fitted in each model [related to the main analysis and Figure 1B].

Table S1

**Variable**	**Deviance (χ^2)**	***df***	***P***
**Cues visible**	4.38	2	0.11
**Model**	0.55	1	0.46
**Cues visible*Model**	7.03	2	0.03*
Stimuli direction	0.62	1	0.86
Centre	2.54	6	0.85
Age	0.22	1	0.64
Sex	0.33	1	0.57
Sex*Age	1.20	2	0.55

Supplemental Results

Interaction between cues visible and model

The model that best fitted the data contained cues visible, model horse viewed, and the interaction between these variables (Table S1). This model fitted the data significantly better than the null hypothesis, χ^2 (6) = 14.70, P=0.02. The interaction in this model reveals that sensitivity of accuracy to cues visible differed according to the model horse subjects were looking at. Fisher's Exact Tests demonstrated that when viewing MC subjects had a higher accuracy rate when the eyes were covered (ears visible) than when viewing WG (MC 83% vs WG 33% accuracy, P=0.04). However, there was no difference when the ears were covered (MC 42% vs WG 58% accuracy, P=0.68) or when all cues were visible (MC 67% vs WG 83% accuracy, P=0.64).

Time spent looking at the photograph and latency to approach a bucket

Horses looked at the photograph for longest when the whole head was uncovered (*M*=3.21s, *SEM*=0.91) and looked for less time when the eyes and ears were covered (eyes: *M*=1.52s, *SEM*=0.48; ears: *M*=1.14s, *SEM*=0.21; see main text for statistical comparison). None of the variables of interest (cues visible, model horse seen, and congruency of choice) influenced latency to approach a bucket (P>0.05 for all comparisons).

Supplemental Experimental Procedures

Study animals

A total of 82 horses were tested and of these 72 were included in the final analysis. Reasons for exclusion were: methodological error (2); approached the set up at an oblique angle (5); horses chose not to participate (3). Of the 72 horses included in the final analysis, 52 horses were geldings and 20 mares. Ages ranged from 3 – 30 years (*M* =14.97, *SD* = 5.90). Subjects had no known eyesight

problems. Horses were from several yards in the South-East and North-West of England and were housed under various conditions. This ranged from horses that were kept outside all year round to horses that were stabled 24 hours a day during winter and turned out in the summer. Some horses lived with between 0 and 6 regular companions, whereas others were turned out with groups that varied in size and composition. All horses had experience of a field companion wearing fly masks; however, only 7 horses had personal experience of actually wearing the masks. All testing was completed in the indoor arena on the yard where the participant resided.

Training trials

All horses participated in one training trial. A bucket containing 20g of concentrated hard feed was placed in front of a plastic block at the edge of an indoor arena (Figure S1B). Horses were led into the arena and walked in a figure of eight before being turned towards the training set up. Horses were released at a point (R) 5m away from the apparatus to approach the bucket and receive the food reward (Figure S1A). If the horses did not approach then the experimenter encouraged them. Once the horse had eaten it was caught and lead away while the experimental apparatus was set up. If the horse did not chose to approach and eat from the bucket it did not move forward to the test trial.

Test trials

Once the horse had been lead away, a second person set up the test trial so the experimenter leading the horse was blind to the sequence of presentation. The photographic stimulus was attached to a wall, and a wooden pole was placed perpendicular to the wall, with one end raised up on a block directly under the photograph (Figure S1C). The pole was to encourage the horses to make an unambiguous choice. One bucket was placed 150cm either side of the dividing pole. Both buckets contained an identical food reward (20g concentrated food) to remove the possibility of horses using odour to locate the food. Once the test apparatus was set up the horse was brought back and led in

another figure of eight before turning in and being released at point R, 5m away from the photograph (Figure S1A). Upon release the experimenter leading the horse walked away and stood directly behind the horse facing away from the experimental set up (point E). This was done to make the experimenter blind to the horses' responses, and to remove the opportunity for the horse to pick up on incidental cues (the Clever Hans Effect). In addition to this the experimenter was blind to the sequence of presentation, was instructed to look at the floor during leading, and wore a peaked cap to help obscure the view of the photograph. Horses were given two minutes to approach the set up and make a choice. If they did not approach within this time (or wandered away from the trial) then they were caught, walked in another figure of eight, and released again. Horses that did not make a choice after 3 releases were counted as non-responders.

Stimuli

Photographs were taken of horses looking at a bucket of food on the floor 150cm away. Two horses were used as the models; a bay Irish draft x thoroughbred gelding (WG) and a black Dutch sports horse mare (MC). The models were unfamiliar to the participants, to avoid potential confounding effects of dominance. Photographs were taken with a Canon 400D DSLR camera. Two photographs of each horse were selected (one looking left and one looking right) to create the final stimuli set. The images were then extracted, placed onto a uniform white background, and auto adjusted for levels and brightness in Adobe Photoshop. Further stimuli were created by adding eye and ear masks with Photoshop to obscure the view of the eyes or ears. These masks are commonly used for horses that are sensitive to flies, and the nature of the fabric means that although horses can see out of them, the external view of either the eyes or ears is obscured. This created six stimuli of each model horse (whole head left and right; eyes covered left and right; ears covered left and right – see main text for a visual representation). The stimuli were enlarged to A1 (841 x 594 mm) and printed.

Stimuli Validation

Sheep, a domestic ungulate with similar vision to horses, show remarkable abilities for recognising the faces of other sheep in photographic stimuli (for an example see [S1]). Consequently it was expected that horses would also be able to recognise the photographic stimuli developed for this experiment as depicting a horse, even when key parts of the face were covered. If the horses did not recognise the photographs, we would expect them to react to the stimuli as they would to any other comparable novel object.

To explore this, we compared reactions to the stimuli to reactions to an appropriately matched novel object in a separate experiment. The novel objects used were matched, phase-scrambled images of the stimuli. Phase-scrambled images contain the same physical energy properties as the original image, but are rearranged so none of the original facial features are distinguishable; making them an ideal control for recognition tasks (personal communication, Graham Hole; see [S2] for an example). The phase-scrambled images were generated in Matlab R2010a.

24 horses (aged from 11–21 years, M = 16.95, SD = 2.97; 20 geldings, 4 mares) participated in a mixed design. Each horse saw one standard version of a stimulus and the corresponding scrambled version in separate presentations. Time between presentations was at least 7 days (M=23.63, SD=19.27) and the first presentation varied equally between the standard and scrambled stimuli. Stimuli were presented for a total of 30 seconds: An experimenter initially held the stimuli up at a point 60cm from the horse's nose for 10 seconds, then moved it forward approximately 10cm and held it there for 10 seconds, before moving it back to the starting position for a final 10 seconds. A second experimenter held the horse on a loose rope, facing the rear of the horse so she could not see the procedure and incidentally cue the horse. Trials were video recorded, and time spent looking at the stimuli, time spent avoiding the stimuli, and time spent approaching the stimuli were measured.

One-way ANOVAs demonstrated that the horses appraised the three standard stimuli in a similar way. There was no significant difference in avoidance behaviour, approach behaviour, or looking behaviour across the three conditions of standard stimuli, $p>0.05$ for all comparisons; as these horse stimuli did not elicit different responses, the following comparisons reflect responses to the three conditions of standard stimuli - all cues visible, eyes covered, and ears covered - combined in comparison with the control stimuli. Importantly, horses reacted significantly differently to the standard stimuli than to the scrambled control stimuli. Horses were significantly more likely to approach the horse stimuli (M=7.60, SEM= 1.49) than the control stimuli (M=3.83, SEM=1.63), $t(23)=2.45$, $p=0.02$. In addition, horses were significantly more likely to avoid the scrambled stimuli (M=13.67, SEM=2.25) than the horse stimuli (M=0.82, SEM=0.33), $t(23)=5.98$, $p<0.001$. There was no difference in looking time between the horse stimuli (M=17.78, SEM=1.42) and the scrambled stimuli (M=14.84, SEM=1.57), $t(23)=1.33$, $p>0.05$. From these comparisons we concluded that subjects were able to recognise the photographic stimuli as depicting horses, even when the masks covered some of the internal features of the face.

Ethical statement

The study was reviewed and approved by the University of Sussex Ethical Review Committee. The Association for the Study of Animal Behaviour Guidelines for the Treatment of Animals in Behavioural Research and Teaching were adhered to. Owners/care takers of the horse gave consent prior to participation. Horses were not food deprived and remained in their familiar environment during participation in the study.

Video analysis

All videos were analysed frame by frame using Sportscode Gamebreaker 7.5.5. Coding was performed by JW, with 15% (11/72) of videos for the main experiment and 17% (8/24) of videos for the validation trials coded by a second observer. Cronbach's alpha revealed good to excellent levels of agreement for the measures of interest: bucket chosen to feed from (1); latency to approach (1); time looking at photograph (0.82); time avoiding the stimuli (0.87); time approaching the stimuli (0.87). The video analysis for the main trials was performed blind to the experimental condition.

Statistical analysis

The main behaviour studied was which bucket the responding horse chose to feed from, and whether the bucket was congruent or incongruent with the bucket the model horse was looking at. Accuracy was assessed using two-tailed binomial tests.

To assess any potential influence of age and sex of the horse, which centre the horse was tested at, the model horse, and the direction the stimuli pointed, these variables were entered along with condition/cues visible as predictors in a logistic regression. Choice was used as the binary response variable (0 = choice incongruent with model, 1= choice congruent with model). Whether inclusion or exclusion of parameters in the model significantly improved the fit was tested by comparing the difference between the deviance values of two models, which are distributed approximately as chi-squared (χ^2) with degrees of freedom (df) equivalents to the difference in the number of parameters fitted in each model [S3].

The potential influence of cues visible, model horse seen, and congruent choice on the latency to approach and time looking at the photograph (measured in seconds) were analysed using a 3-way independent ANOVA. Planned comparisons were used to follow up significant results.

Supplemental References

S1. Tate AJ, Fischer H, Leigh AE, Kendrick KM (2006) Behavioural and neurophysiological evidence for face identity and face emotion processing in animals. Philosophical transactions of the Royal Society of London Series B, Biological sciences 361: 2155-2172.

S2. Bukowski H, Dricot L, Hanseeuw B, Rossion B (2013) Cerebral lateralization of face-sensitive areas in left-handers: Only the FFA does not get it right. Cortex 49: 2583-2589.

S3. Dobson AJ (2010) An Introduction to Generalized Linear Models, Second Edition: Taylor & Francis.
